# Supplementary material for: Activation of 5-HT7 receptors in the mouse dentate gyrus does not affect theta-burst-induced plasticity at the perforant path synapse
Source: Pharmacol Rep. 2024 Nov 2;76(6):1377–89. doi: 10.1007/s43440-024-00674-6 (PMC11582198; doi:10.1007/s43440-024-00674-6)
Supplement: Supplementary file 1 — Supplementary file1 (PDF 2368 KB) [file 43440_2024_674_MOESM1_ESM.pdf]

Supplementary Figures

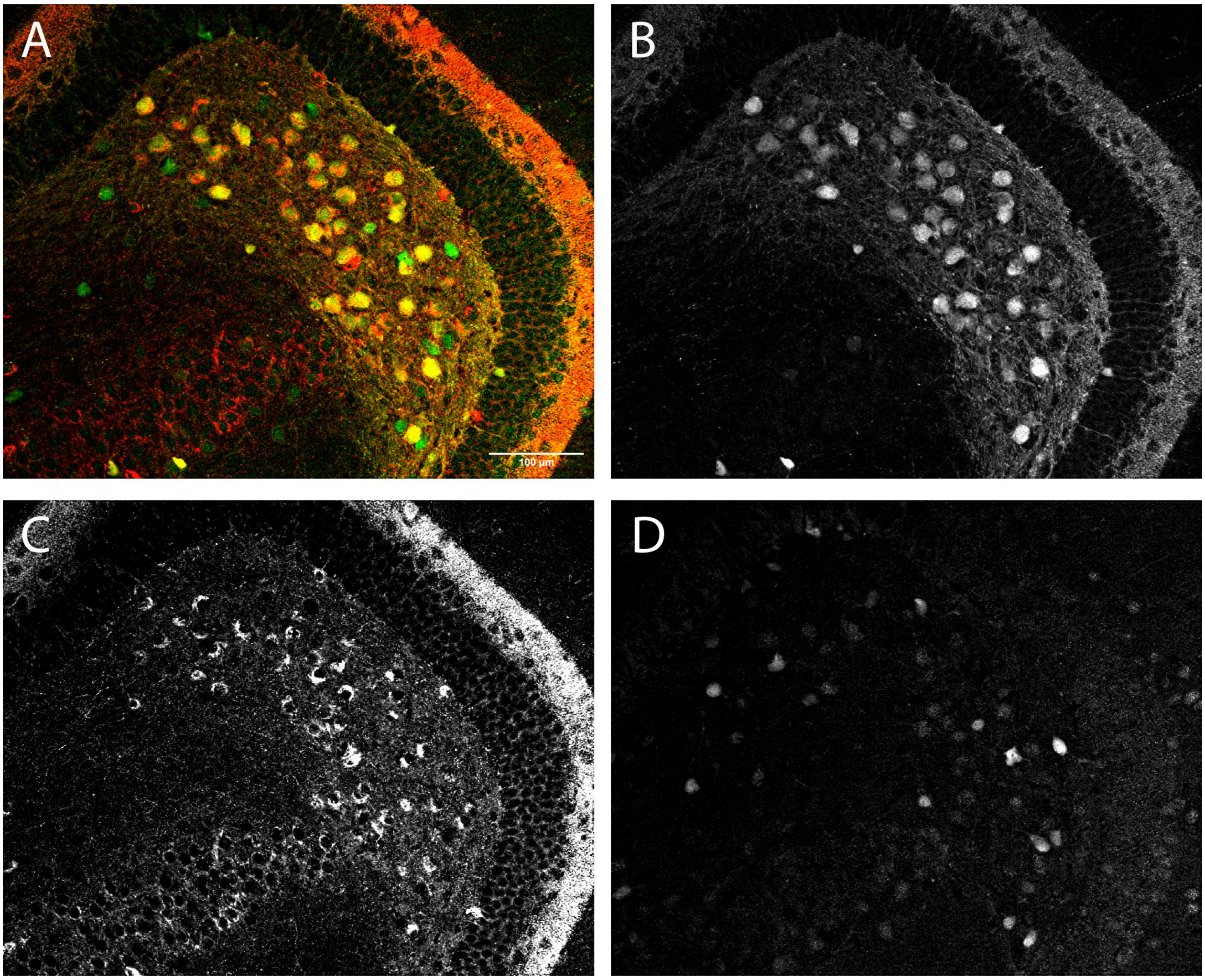

Figure 5. (A) Representative composite frame image of a mouse dentate gyrus section stained for calretinin (CR), pro-cholecystokinin (pCCK) and GFP. (B-D) Single channels from image A corresponding to CR (panel B), pCCK (C) and GFP (D).

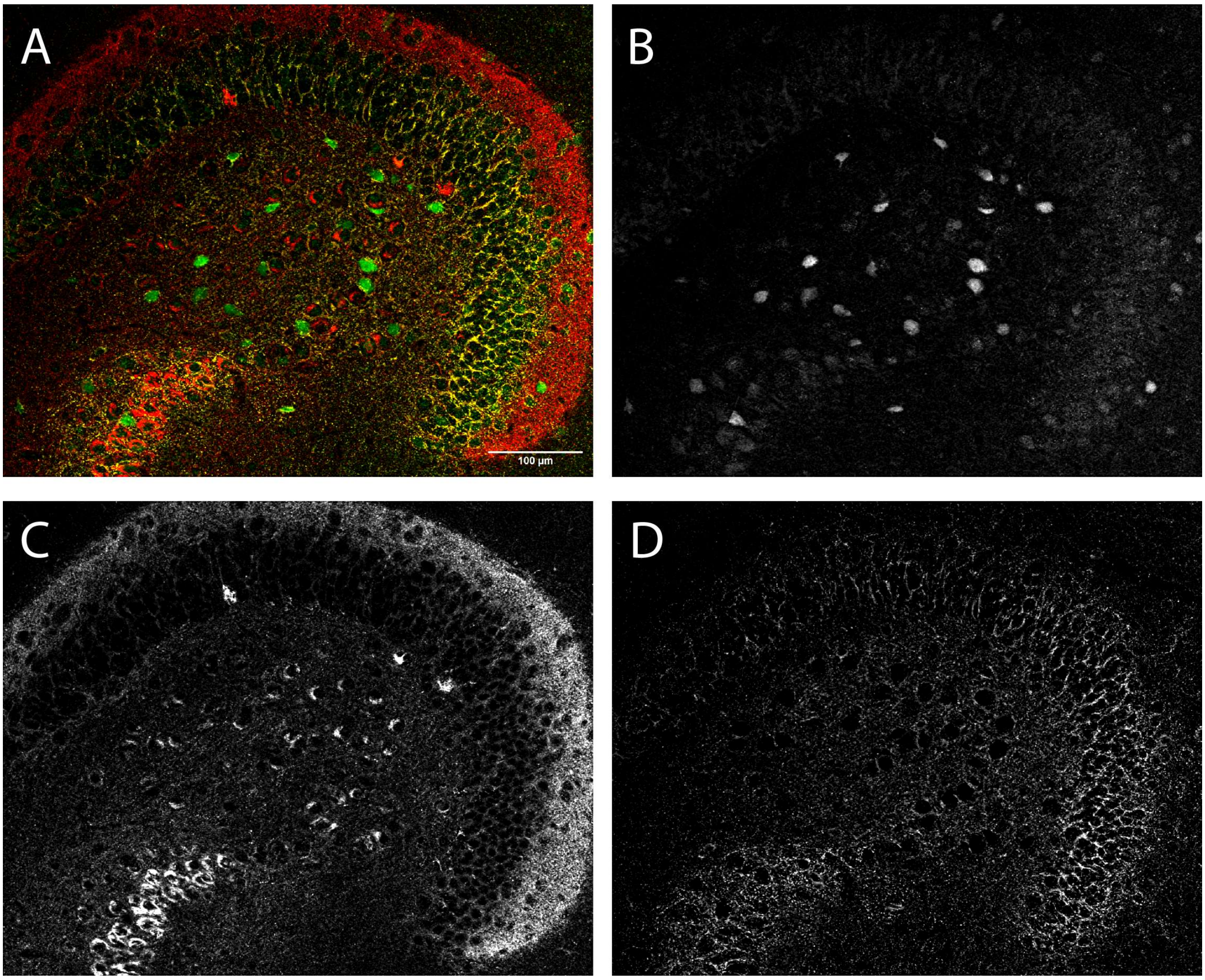

Figure 6. (A) Representative composite frame image of a mouse dentate gyrus section stained for GFP, pCCK and glutamate decarboxylase (GAD67). (B-D) Single channels from image A corresponding to GFP (panel B), pCCK (C) and GAD67 (D).
